# Haematological Profile in Patients With Acute Falciparum Malaria: A Hospital-Based Study

**DOI:** 10.7759/cureus.63690

**Published:** 2024-07-02

**Authors:** Somnath Roy, Debashree Roy Saha, Rashmi Ahmed, Narayan C Sharma, Putul Mahanta

**Affiliations:** 1 Internal Medicine, Gauhati Medical College and Hospital, Guwahati, IND; 2 Dermatology, Nalbari Medical College and Hospital, Nalbari, IND; 3 Community Medicine, Lakhimpur Medical College, Lakhimpur, IND; 4 Pediatrics, PA Sangma International Medical College and Hospital, Khanapara, IND; 5 Forensic Medicine and Toxicology, Nalbari Medical College and Hospital, Nalbari, IND

**Keywords:** severe malaria, bone marrow aspiration, erythropoietic response, thrombocytopenia, anaemia, haematocrit

## Abstract

Introduction

Malaria is the most common parasitic disease affecting humans. Haematological alterations in malaria are expected, and these changes play a significant role in fatal complications. The present study aims to assess the clinical and haematological profile in patients with acute falciparum malaria and the significance of various haematological and coagulation alterations with the clinical severity of malaria.

Methods

The prospective cross-sectional study included 68 acute falciparum malaria cases. Thick and thin blood film microscopy and a rapid diagnostic kit were used to diagnose malaria. The cases were subjected to various haematological and biochemical investigations. Bone marrow aspiration samples were also collected. Using appropriate statistical methods, the findings were compared between severe and uncomplicated malaria cases. A p-value below 0.05 was considered significant.

Results

The participants’ ages ranged from 14 to 78. Most participants (n = 51, 75%) were male and belonged to the lower income group (33, 48.5%). Significant variations in mean parasite count between severe and uncomplicated malaria cases (p-value < 0.01) were observed. The severe and uncomplicated groups showed significant differences in haemoglobin (gm/dL), haematocrit, red blood cell count, reticulocyte, serum iron, and ESR levels (p-value < 0.05). The severe malaria group had considerably reduced mean platelet counts (p-value < 0.01). Only five instances (7.3%) had an appropriate erythropoietic response after day 28. Erythroid hyperplasia with dyserythropoietic alterations was most common in patients with severe anaemia and low-grade parasitaemia.

Conclusion

Acute falciparum malaria is often associated with haematological alterations. Anaemia and thrombocytopenia were the most expected alterations associated with disease prognosis and mortality.

## Introduction

Malaria is the most significant parasite illness affecting humans. Blood-feeding female anopheline mosquitoes bite humans, transferring a protozoan infection of red blood cells (RBCs). *Plasmodium falciparum*, *P. vivax*, *P. ovale*, *P. malariae*, and *P. knowlesi* are the five most involved species of *Plasmodium* genus of parasitic protozoans causing human malaria [1]. The erythrocytic schizogony in the blood is the source of all malarial clinical manifestations [2]. The immunological condition of the host in the past affects the clinical symptoms. There is a semiquantitative correlation between the risk of death and the level of parasitaemia, particularly in nonimmune expatriates infected with *P. falciparum*. Without prompt treatment, *P. falciparum* malaria in nonimmune individuals can escalate quickly to severe malaria [3].

South-east Asia contributes 2.5 million cases of malaria, out of which 76% constitutes from India alone [4]. In Assam, malaria is a serious public health concern that affects 30-40% of the population at high risk. Malaria transmission is perennial and chronic in most sections of the state despite decades of attempted control initiatives. There is a resurgence of malaria outbreaks correlated with elevated *P. falciparum* levels and occurrences of preventable mortality [5].

Haematological alterations in malaria are expected as they are blood parasites for most of their intricate life cycle. Malaria immunity, baseline haemoglobinopathy, endemicity level, and demographics all contribute towards the haematological changes linked to malaria. Anaemia, thrombocytopenia, leucocytosis, or leucopenia are the common haematological alterations observed among malaria patients [6]. Although the exact aetiology of thrombocytopenia in malaria remains unknown, it is thought that the disease causes accelerated obliteration of platelets and decreased platelet lifespan, resulting in noticeable splenomegaly and circulated immunological complexes [7]. Malaria has been found to have a general effect on anaemia and other haematological variables. This is especially problematic since recurring malaria episodes can induce life-threatening anaemia and metabolic acidosis, especially in youngsters [8]. Studies also suggested that haematological changes in severe malaria vary with the development of complications [9]. In malaria infection, the bone marrow plays a vital role in host-pathogen interactions [10]. Both *P. falciparum* and *P. vivax* malaria include a range of abnormalities in blood and bone marrow cell counts, appearance, and function. The type of these anomalies in a nonimmune person depends on the duration since the infection occurred. In others, it depends on the degree of host immunity and the pattern and severity of malaria transmission in the region [11]. By restricting blood circulation, cytoadherence and rosetting contribute to malaria pathophysiology by lowering oxygen levels in tissues, creating excessive lactate, and depressing blood and tissue pH, thereby increasing the risk of severe anaemia [11]. Haematological parameters can help determine a tentative course of treatment, particularly in situations when the findings of the parasitological test are ambiguous or not readily available. Thus, anticipating the haematological alterations associated with malaria might help to implement a successful and prompt treatment plan to avert grave consequences [7].

Along with the resurgence of malaria in northeast India, growing issues with drug-resistant parasites and insecticide-resistant vectors, which cause hyperparasitaemia and the emergence of significant systemic consequences, need serious attention. Although haematologic changes play a significant role in these fatal complications, there was insufficiently detailed research on the clinical-haematological changes in falciparum malaria from northeast India.

Therefore, the present study aims to assess the clinical and haematological profile in patients with acute falciparum malaria and the significance of various haematological and coagulation alterations with the clinical severity of malaria. Also, the study aims to investigate the probable correlation between haematological and coagulation abnormalities and the degree of parasitaemia.

## Materials and methods

The prospective cross-sectional study was conducted at the Department of Medicine, Gauhati Medical College and Hospital, Guwahati, India, from March 2018 to February 2019. Patients above 12 years of age admitted with clinically suspected “malaria” having asexual forms of *P. falciparum* in peripheral blood smears and consenting to physical examination, investigations, and four consecutive weekly follow-up evaluations were included. Analysis of symptoms and haemogram parameters was done in all these patients. The current study has ethical approval from the Gauhati Medical College, Guwahati Ethics Committee, vide ref: EC/MC/GMC/150. Informed consent was obtained from all the study participants who agreed to participate voluntarily. The study was carried out in compliance with the Declaration of Helsinki, ensuring the confidentiality of the study participants.

Patients above 12 years of age were admitted to the study hospital with clinically suspected “malaria” having asexual forms of *P. falciparum* in the peripheral blood smear included. Patients less than 12 years of age, pregnant women with severe malnutrition, mixed malarial infection, or having other coincident infection or parasitic infestation were not included in the study to limit the potential influencing effects of those conditions on haematological parameters. Patients on chemotherapeutic drugs during the preceding two weeks were also excluded. Patients with medical history indicating pre-existing systemic diseases to which haematological alterations can be attributed or had haemoglobinopathy, had recrudescence of parasitaemia during the four weeks of follow-up, or had a blood transfusion during the study period were also excluded.

The selected patients were divided into two groups, severe malaria and uncomplicated malaria, to facilitate the subsequent assessment of haematological abnormalities. The severe malaria group consisted of cases of acute falciparum malaria with complications. Changes in sensorium, repeated generalised convulsions, raised serum creatinine with or without oliguria, dyspnoea with changes in chest X-ray or abnormal chest findings, severe anaemia, jaundice, hypoglycaemia, shock, acidosis, haemoglobinuria, and spontaneous bleeding with or without laboratory evidence of DIC were taken as indications of systemic complications as per WHO criteria for severe malaria [12]. At the same time, the uncomplicated malaria group included cases of acute falciparum malaria without complications. A detailed medical history was obtained in all cases, and a complete physical examination was carried out on admission and at least once a day. Comprehensive records were kept about the course of the disease, treatment given, and any complications that developed.

Non-microscopic rapid malaria diagnostic tests (ParaSite-F test/ICT Malaria Pf) and thick and thin blood smears were used to detect and confirm malarial parasites. Blood from a finger prick was used to generate both thick and thin blood films on a slide. The thin film was fixed with pure methyl alcohol (acetone-free) for three to five minutes. Then, the blood film was stained with 10% Giemsa stain. The slides were examined under oil immersion to detect parasite presence and determine species and parasite count. If parasites were not found with one or other, the staining process was repeated for a second or third time at six hours.

The cases were then subjected to various haematological investigations, including a complete blood count for RBC and white blood cell (WBC) indices, peripheral blood smear examination for RBC morphology, and patient coagulation profiles. Biochemical investigations included glucose-6-phosphate dehydrogenase assay, serum iron, folate and total iron binding capacity, blood sugar estimation, urine and stool examination, renal function tests for serum creatinine and blood urea, liver function tests for serum bilirubin and its fractions, serum protein and its fractions, and SGPT. Normal values (reference ranges) for the haematological findings were based on recommendations by Dacie and Lewis [13]. Parasite concentration was estimated every 12 hours until the patient appeared aparasitaemic and then daily. The haematological and biochemical investigations were repeated on days 7, 14, and 28 after initiation of therapy. All patients underwent chest X-ray, electrocardiogram, and funduscopic examination.

Bone marrow aspirate samples were collected from the posterior iliac spine using Salah and Klima needles. Bone marrow films were fixed and stained with Romanowsky dyes. The slides were then examined for free malaria parasites, phagocytosed plasmodia, free haemozoin pigments, pigmented leucocytes, iron stores, differential count, cellularity, erythropoiesis, myeloid series, lymphoid elements, plasma cell elements, megakaryocytic series, and dyserythropoietic features, mostly irregularly shaped nuclei and karyorrhexis.

Parasite density

The parasite density was recorded as follows:

+: one to 10 parasites per 100 oil immersion fields

++: 10 parasites per 100 oil immersion fields

+++: one to 10 parasites per one oil immersion field

++++: >10 parasites per one oil immersion field

Parasite count

Thick Film

Infected erythrocytes are counted about a predetermined number of WBCS, and an average of 8000/µL is taken as standard. Two hundred leucocytes are counted in 100 fields (0.25 µL of blood).

If >10 parasites are counted: (no. of parasites/no. of WBCS counted) × 8000 = no. of parasites/µL.

If 200 leucocytes are counted: no. of parasites counted × 40 = no. of parasites/µL.

If the parasites are less than nine, then count 500 WBCS: no. of parasites counted × 16 = no. of parasites/µL.

Thin Film

To determine the percentage of parasitaemia of *P. falciparum*, the number of infected red cells (and not the number of parasites) in 1000 RBCs is converted to percentage. The approximate number of parasites present in 1 µL of blood can be calculated by assuming that 1 µL of blood contains 5 × 10 RBC; therefore, a 1% parasitaemia will contain one parasite/100 RBC or 50,000 parasites/µL of blood.

Statistical analysis

All the data were recorded in a structured proforma. IBM SPSS Statistics, version 21.0 (IBM Corp., Armonk, NY) was used to analyse the data. The haemogram parameters of severe and uncomplicated malarial groups were compared using the student’s unpaired t-test or non-parametric counterpart. The correlation between parasitaemia and different haematological parameters was also evaluated using Pearson’s correlation coefficient or Spearman’s correlation coefficient, depending on the normality of the data. A p-value below 0.05 was considered statistically significant.

## Results

The study included 68 falciparum malaria patients for clinical manifestations and haematological changes. The age of the participants ranged from 14 to 78 years, with a mean (±standard deviation) of 36.8 (±14.9) years, mostly belonging to the third and fourth decade (n = 38, 55.9%) of life. A majority (n = 51, 75%) of the participants were male and belonged to lower (n = 33, 48.5%) and middle (44.1%) income groups (Table 1).

**Table 1:** Socio-demographic distribution of the study participants The data has been represented as the frequency of patients (n) and percentage (%).

Parameters	Categories	Number of cases	Percentage
Age-group	≤20 years	7	10.3
	21-40 years	38	55.9
	41-60 years	16	23.5
	Above 60 years	7	10.3
Sex	Female	17	25.0
	Male	51	75.0
Income status	Higher (above 25,000 rupees per month)	5	7.4
	Middle (10,000-25,000 rupees per month)	30	44.1
	Lower (<10,000 rupees per month)	33	48.5

The frequent exposure of the male population to mosquito resting areas for outdoor activities and poor utilisation of preventive and control measures among the economically poorer group might contribute towards the high incidence of the disease among these groups.

**Figure 1:**
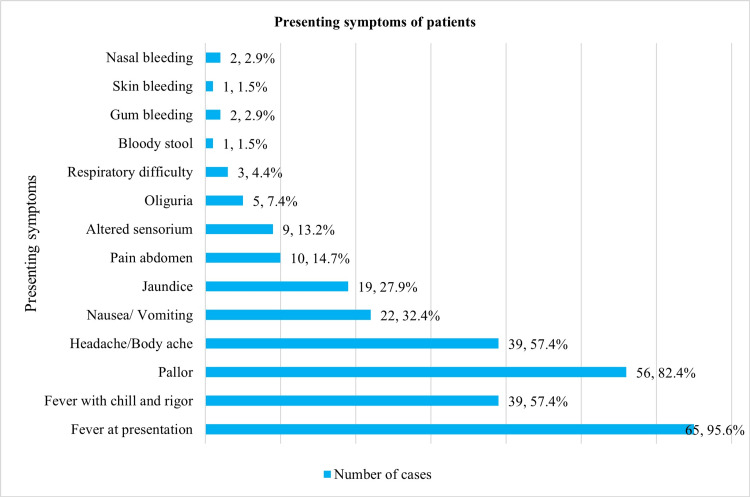
Presenting symptoms of patients

Fever (n = 65, 95.6%) with or without chill and rigour was the main presenting symptom among the patients. Prominent symptoms of severe malaria among the participants included jaundice (n = 19, 27.9%), pain in the abdomen (n = 10, 14.7%), altered sensorium (n = 9, 13.2%), oliguria (n = 5, 7.4%), respiratory difficulty (n = 3, 4.4%) and bleeding (n = 5, 8.8%). On examination, pallor was observed in 56 (88.2%) cases (Figure 1).

The most critical symptoms related to severe malaria observed among the patients were jaundice, pain in the abdomen, altered sensorium, spontaneous bleeding, and oliguria.

Most patients (75%, n = 51) presented during the first week of fever. The pattern of fever was irregular in 35 (51.5%) patients. Continuous fever was observed in 12 (17.6%) patients. At the same time, the rest of the patients were observed to have intermittent fever, mostly with tertian type (23.5%, n = 16) (Table 2).

**Table 2:** Duration and pattern of fever of the patients The data has been represented as the frequency of patients (n) and percentage (%).

Clinical manifestations	Categories	Number of cases	Percentage
Duration of fever at presentation	0-2 days	5	7.4
	3-5 days	19	27.9
	6-8 days	27	39.7
	9-11 days	9	13.2
	12 days onwards	8	11.8
Pattern of fever	Continuous	12	17.6
	Quotidian	5	7.4
	Tertian	16	23.5
	Irregular	35	51.5

Organomegaly was observed in 67 (98.5%) cases, among whom more than one-third (n = 23, 33.8%) had hepatosplenomegaly. Hepatomegaly was detected in 24 (35.3%) cases, and splenomegaly was found in 20 (29.4%) cases. Multiple organ dysfunction was recorded in four out of 68 patients. All four cases were observed to have high parasite density with haematological abnormalities and other systematic complications.

**Figure 2:**
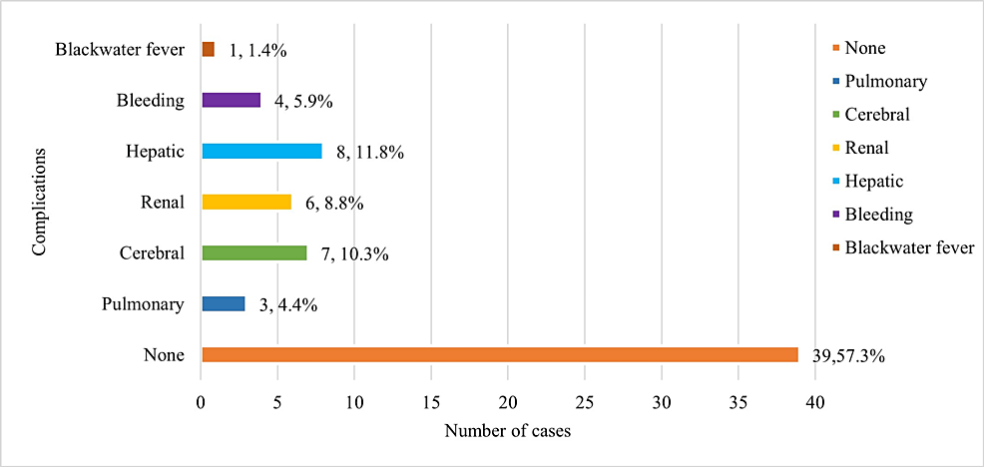
Systematic manifestations The data has been represented as the frequency of patients (n) and percentage (%).

Out of the 68 patients, 29 (42.6%) patients had severe acute falciparum malaria with complications. The major complications observed among the patients were hepatic (11.8%, n = 8), cerebral (10.3%, n = 7), and renal (8.8%, n = 6) involvements (Figure 2).

Table 3 shows a significant difference between severe and uncomplicated groups for mean parasite count percentage (p-value < 0.01). Also, various haemogram parameters differed substantially between the severe and uncomplicated groups. The mean haemoglobin (gm/dL) level was 8.7 ± 1.8 gm/dL in the severe malaria group and 10.8 ± 1.4 gm/dL in the uncomplicated malaria group, and the difference was highly significant (p-value < 0.01). Similarly, the mean haematocrit percentage (27.0 ± 6.9 vs. 33.1 ± 4.4; p-value < 0.01) and mean RBC count (2.9 ± 0.8 vs. 3.7 ± 0.5; p-value < 0.01) were significantly lower in the severe malaria group as compared to the uncomplicated group. Mean reticulocyte count percentage (2.1 ± 0.9 vs. 1.7 ± 0.4; p-value < 0.05), mean serum iron levels (11.2 ± 1.9 µmol/L vs. 9.6 ± 2.3 µmol/L; p-value < 0.01), and mean ESR levels (39.2±6.6 vs. 34.4±5.9; p-value<0.01) were observed to be higher in the severe malaria group. Although the mean WBC count does not differ significantly between the two groups, the mean platelet count (75.3 ± 35.8 × 10^3^/mm^3^ vs. 110.6 ± 25.6 × 10^3^/mm^3^; p-value < 0.01) was observed to be significantly lower in the severe malaria group. The severe malaria group also exhibited higher mean bleeding and clotting time (p-value < 0.01) than the uncomplicated malaria group.

**Table 3:** Parasite count and haematological variations in severe and uncomplicated falciparum malaria cases The data has been represented as mean and standard deviation (std dev). *p-value < 0.05; the difference is statistically significant.

Haematological parameters	Uncomplicated malaria (n = 39)	Severe malaria (n = 29)	t-test	p-value
	Mean ± std dev	Mean ± std dev		
Parasite count (%)	0.06 ± 0.07	13.6 ± 14.9	-5.7	<0.01*
Haemoglobin (gm/dL)	10.8 ± 1.4	8.7 ± 1.8	5.41	<0.01*
Haematocrit (%)	33.1 ± 4.4	27.0 ± 6.9	4.38	<0.01*
Red blood cells (×10^12^/L)	3.7 ± 0.5	2.9 ± 0.8	4.81	<0.01*
Reticulocyte (%)	1.7 ± 0.4	2.1 ± 0.9	-2.45	0.02*
Serum iron (µmol/L)	9.6 ± 2.3	11.2 ± 1.9	-2.97	<0.01*
White blood cells (×10^3^/mm^3^)	6.8 ± 2.2	8.2 ± 3.1	-2.01	0.05
Platelet (×10^3^/mm^3^)	110.6 ± 25.6	75.3 ± 35.8	4.52	<0.01*
Bleeding time (min)	5.1 ± 0.6	6.5 ± 1.3	-5.84	<0.01*
Clotting time (min)	7.5 ± 0.8	9.5 ± 1.8	-5.96	<0.01*
Erythrocyte sedimentation rate (mm AEFH)	34.4 ± 5.9	39.2 ± 6.6	-3.14	<0.01*

No significant correlation was obtained between malaria parasitaemia and various haematological parameters in uncomplicated malaria. In severe malaria, the patient’s age (correlation-coefficient, r = 0.57; p-value < 0.01) was positively correlated with parasitaemia. A significant positive correlation between malaria parasitaemia and WBC levels indicates that per unit rise of malaria parasitaemia results in a 0.54 unit rise in WBC levels (p-value < 0.01). Also, the significant negative correlation of malaria parasitaemia with haemoglobin (correlation-coefficient, r = −0.37; p-value < 0.05) and platelet levels (correlation-coefficient, r = −0.46; p-value < 0.05) indicates considerable declinations in those haematological features with per unit rise in parasitaemia. Malaria parasitaemia had a significantly positive correlation with prothrombin time (p-value < 0.05). It activated partial thromboplastin time (p-value < 0.05 in severe malaria), indicating a substantial impact of malaria parasitaemia on coagulation anomalies (Table 4).

**Table 4:** Major correlation of malaria parasitaemia with various haematological parameters among uncomplicated and severe falciparum malaria cases *p-value < 0.05; the difference is statistically significant. **p-value < 0.01; the difference is statistically highly significant.

Parasite density	Uncomplicated malaria (n = 39)		Severe malaria (n = 29)	
	correlation co-eff (r)	p-value	correlation co-eff (r)	p-value
Age	0.07	0.69	0.57	<0.01**
Haemoglobin (gm/dL)	0.21	0.20	-0.37	0.048*
White blood cells (×10^3^/mm^3^)	0.26	0.11	0.54	<0.01**
Platelet (×10^3^/mm^3^)	-0.05	0.77	-0.46	0.01**
Prothrombin time (min)			0.38	0.04*
Activated partial thromboplastin time (min)			0.38	0.04*

The mean haemoglobin concentrations, haematocrits, and reticulocyte counts decreased during the first week after initiation of treatment, returned to baseline after day 14, and then gradually increased until day 28, except for reticulocyte count, which showed a fall. The low mean platelet counts returned to normal in all the patients by day 7, and the mean granulocyte counts were normal throughout the observation period. The erythrocyte sedimentation rate recorded a progressive decline in both the malaria groups throughout the follow-up period (Table 5).

**Table 5:** Comparison of haematological and biochemical profiles of patients during the four weeks of follow-up The data has been represented as the mean of parameters.

Parameter	Type of malaria	Day 0	Day 7	Day 14	Day 28
Blood count					
Haemoglobin (gm/dL)	Uncomplicated	10.8	10.0	10.2	12.6
	Severe	8.7	7.8	8.4	9.0
Haematocrit (%)	Uncomplicated	33.1	31.4	32.9	34.2
	Severe	27.0	24.8	26.2	29.5
White blood cells (×10^3^/mm^3^)	Uncomplicated	6.8	5.2	5.0	5.4
	Severe	8.2	6.4	5.9	6.6
Platelet (×10^3^/mm^3^)	Uncomplicated	110.6	290	284	275
	Severe	75.3	305	323	267
Markers of inflammation					
Erythrocyte sedimentation rate (mm AEFH)	Uncomplicated	34.4	27	20	18
	Severe	39.2	33	29	25
Albumin (gm/dL)	Uncomplicated	4.0	4.1	4.1	4.4
	Severe	3.1	3.2	3.2	3.3
Measures of erythrocyte production and destruction					
Reticulocyte (%)	Uncomplicated	1.7	1.5	1.9	1.8
	Severe	2.9	1.6	2.2	2.0
Indirect bilirubin (gm/dL)	Uncomplicated	1.9	0.5	0.7	0.6
	Severe	2.0	1.1	0.9	0.7
Hepatic functions					
Total bilirubin (gm/dL)	Uncomplicated	1.1	0.6	0.8	0.7
	Severe	5.4	1.8	1.1	0.9
SGPT (U/L)	Uncomplicated	38.0	44	36	30
	Severe	61.9	66	59	48
Renal functions					
Serum creatinine (mg/dL)	Uncomplicated	1.3	1.2	1.0	0.9
	Severe	3.3	1.8	1.3	1.1

**Figure 3:**
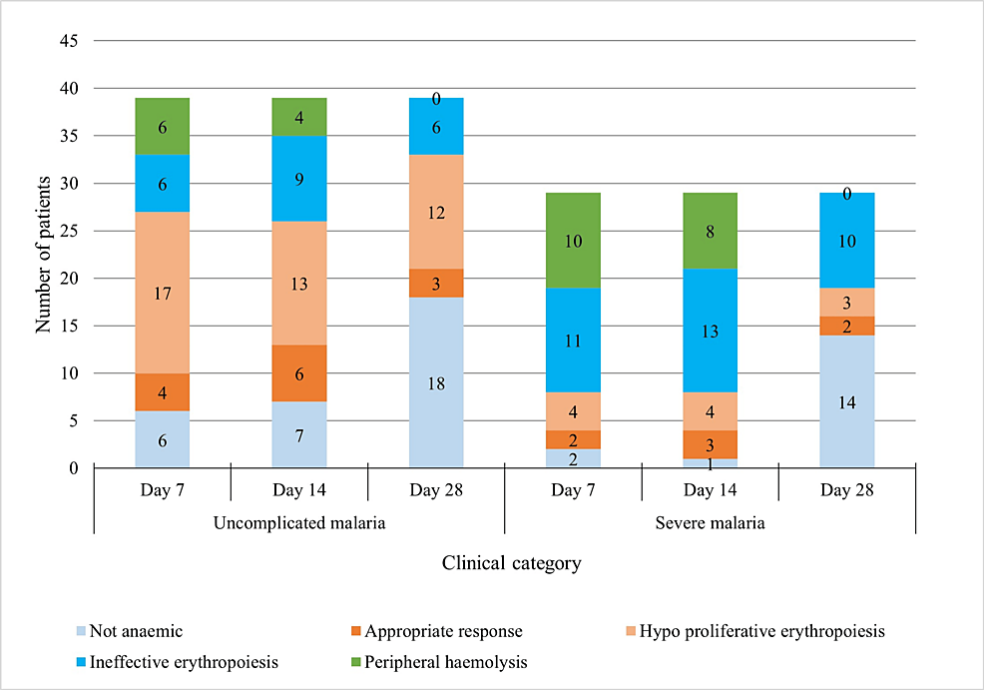
Erythropoietic response after initiation of treatment during the follow-up period among falciparum malaria cases The data has been represented as the frequency of patients (n).

Among the total 68 falciparum malaria cases, the majority of 60 cases (88.2%) had persisting anaemia on days 7 and 14, out of which 36 (52.9%) were still anaemic on day 28. The appropriate erythropoietic response was observed only in five cases (7.3%) after day 28. Hypo proliferative erythropoiesis was most commonly observed among uncomplicated malaria cases, with 17 (25%) cases on day 7 reducing to 12 (17.6%) on day 28. At the same time, ineffective erythropoiesis was most frequent in severe malaria cases, with 10 (14.7%) cases on day 28. Peripheral haemolysis was observed in 16 cases (23.5%) on day 7, which was reduced to 12 cases (17.6%) on day 14. On day 28, none of the cases showed peripheral haemolysis. The erythropoietic response after initiation of treatment during the follow-up period among falciparum malaria cases is shown in Figure 3.

The patients were further divided into three groups to determine the correlation between parasitaemia and the morphological changes observed in the bone marrow. Severe malaria patients with parasitaemia above 4% were subdivided into group I and group II depending on the haemoglobin level threshold of 8 mg/dL. Group III constituted uncomplicated malaria cases with parasitaemia <4% and any haemoglobin level. The bone marrow aspiration findings are shown in Table 6. All bone marrows showed varying degrees of hypercellularity, ranging from mild in group I to gross in group III. Erythropoiesis was mostly normoblastic. Erythroid hyperplasia with dyserythropoietic changes was most common in patients with severe anaemia and low-grade parasitaemia. Iron stores were depleted in most of the cases. In one case, the falciparum parasite was observed in the marrow.

**Table 6:** Bone marrow aspiration findings among the acute falciparum malaria cases The data has been represented as the mean percentage.

Bone marrow aspirate findings	Severe malaria (mean value)		Uncomplicated malaria (mean value)	Normal values
	Group I (Hb > 8 mg/dL, parasite > 4%)	Group II (Hb < 8 mg/dL, parasite > 4%)	Group II (Hb any, parasite < 4%)	
Cellularity (%)	86.8	95.4	97.9	35-65
Megakaryocytes	2.5	2.3	1.7	0-2
Iron stores	Depleted	Depleted	Depleted/normal	
Infiltration/haemoparasites	1 case	Absent	Absent	
Erythroid cells (%)	18.2	30.6	46.5	7-35.5
Erythroblasts with dyserythropoietic features (%)	<10	10-20	>20	2-7
Myeloid cells (%)	37.5	45.3	42.9	45-77
Eosinophils (%)	7.4	3.4	1.7	1.2-5.3
Lymphocytes (%)	37.8	17.9	6.2	11.1-23.2
Monocytes (%)	2.8	1.7	0.8	0-2.5
Macrophages (%)	4.1	2.7	0.6	0-2
Plasma cells (%)	5.3	8.2	2.4	0-3.5

Out of the 68 patients in the present study, eight (11.8%) patients died during the hospital stay. Anaemia and thrombocytopenia were observed in all these patients. Dysfunction of one or more of the critical organs was recorded in all of them.

## Discussion

*P. falciparum* malaria accounts for more than 80% of all malaria cases worldwide. Haematological abnormalities associated with malaria are caused by numerous intricate and poorly understood pathophysiological mechanisms. Predicting the haematological alterations in malaria allows the clinician to initiate an effective and timely therapeutic intervention to avoid serious consequences [7]. To evaluate the clinical and haematological profile in patients with acute falciparum malaria, the significance of various haematological and coagulation alterations with clinical severity of malaria, and their probable correlation with the degree of parasitaemia, the current study included 68 acute falciparum malaria cases attending the study centre.

Most participants belonged to the third (n = 22, 32.4%) and fourth decade (n = 16, 23.5%) of life. Male preponderance (n = 51, 75%) and lower socio-economic status (n = 33, 48.5%) among patients observed in the present study agree with other similar studies [4,14,15]. Fever (n = 65, 95.6%) with or without chill and rigour, headache/body ache (n = 39, 57.4%), nausea/vomiting (n = 22, 32.4%), jaundice (n = 19, 27.9%), and pain abdomen (n = 10, 14.7%) were the major presenting symptoms. Most patients (75%, n = 51) presented during the first week of fever, typically with an irregular fever pattern. Organomegaly was present in 98% of cases. The findings are in agreement with another recent research [15]. As temperature is a key environmental indicator that the parasite uses to coordinate its development in humans, febrile episodes play an essential role in the pathogenesis of malaria [16].

Out of the 68 patients, 29 patients had severe acute falciparum malaria. A substantial difference was noticed between the severe and uncomplicated groups regarding mean parasite count percentage (p-value < 0.01). Furthermore, several haemogram values differed significantly between the severe and uncomplicated groups. The severe malaria group had significantly decreased mean haemoglobin level (gm/dL), haematocrit percentage, and RBC count (p-value < 0.01). Also, severe malaria patients had significantly higher mean reticulocyte count percentage (p-value < 0.05), serum iron levels (p-value < 0.01), and ESR levels (p-value < 0.01). The mean WBC count did not differ substantially between the two groups (p-value = 0.05). However, the severe malaria group had a considerably lower mean platelet count (p-value < 0.01). The coagulation profile of patients showed that the severe malaria group had increased mean bleeding and clotting times (p-value < 0.01) compared to the uncomplicated group. WBC counts (correlation-coefficient, r = 0.54; p-value < 0.01) and patient age (correlation-coefficient, r = 0.57; p-value < 0.01) were shown to be positively correlated with parasitaemia in cases of severe malaria. Additionally, in severe malaria cases, there was a strong negative association found between malaria parasitaemia with haemoglobin (correlation-coefficient, r = −0.37; p-value < 0.05) and platelet levels (correlation-coefficient, r = −0.46; p-value < 0.05). In severe malaria cases, prothrombin time and activated partial thromboplastin time showed a statistically positive connection with malaria parasitaemia.

The high frequency of thrombocytopenia and anaemia and their significant association with *P. falciparum* infection and higher parasitaemia is documented in other recent studies [7,17]. Malaria infection significantly negatively influences haemoglobin levels, particularly among young children. This effect persists even after acute infection, indicating long-term haemoglobin declines even after effective parasite removal [18]. In addition to iron deficiency anaemia and vascular dysfunction, which are partly caused by the attachment of infected RBCs to the endothelium, declines in haematocrit and haemoglobin levels, which are part of pancytopenia and thrombocytopenia, are indicative of Plasmodium infection. In patients with haematologic disorders, the development of inflammation brought on by complement activation and the production of inflammatory cytokines can exacerbate the clinical manifestations of malaria [19]. Platelet indicators are valuable predictors of malaria severity [20]. In cases of malaria infection, thrombocytopenia may result from either inhibition of thrombopoiesis or increased platelet consumption or destruction, or from a combination of the two. Several mechanisms have been proposed to explain platelets’ accelerated clearance or consumption during malarial infection. Recent research concluded that thrombocytopenia is an excellent diagnostic marker of malaria, with 86.3% specificity and 79.5% sensitivity [21]. The effect of malaria severity on coagulation parameters is well documented [22,23]. A high parasitaemia load leads to anomalies in the synthesising and secretion of coagulation factors and their inhibitors and hepatic microcirculation blockage. Moreover, the production of microparticles from RBC macrophages facilitates the general activation of blood coagulation. In severe *P. falciparum* malaria, thrombocytopenia and blood coagulation system activation happen simultaneously, leading to disseminated intravascular coagulation and coagulation failure [24].

Although the follow-up investigations revealed recovery of mean haemoglobin concentrations, haematocrits, and platelet counts to normal levels after 28 days from initiation of treatment, 36 out of 68 cases (52.9%) still had anaemia on day 28. After day 28, the appropriate erythropoietic response was only seen in five patients (7.3%). The majority of cases of uncomplicated malaria showed hypo proliferative erythropoiesis. On the other hand, inadequate erythropoiesis was more common in severe malaria cases. All bone marrows showed varying degrees of hypercellularity. Erythroid hyperplasia dyserythropoietic changes were most common in patients with severe anaemia and low-grade parasitaemia. Severe malaria causes a sharp increase in mortality in patients with anaemia [25]. Malaria-induced haemolysis of infected and uninfected erythrocytes and bone marrow dyserythropoiesis impedes quick recovery from anaemia [25]. Anaemia sets in quickly in severe falciparum malaria because of the high parasite burden. The haemolysis of unparasitised RBCs is the primary cause of this typically abrupt drop in haemoglobin levels. Bone marrow dyserythropoietic continues for several days or weeks after initiation of treatment of acute malaria, resulting in low reticulocyte count during the symptomatic phase of the disease, causing delayed erythropoietic response [25].

Out of 68 patients in the current study, eight (11.8%) died during their hospital stay. Anaemia, thrombocytopenia, and multiple organ dysfunction were present in all of these patients. The degree of parasitaemia in malaria is usually linked to the severity of the illness and the chance of death [26]. Since *P. falciparum* infects RBCs, it typically inhibits high-grade parasitaemia, which results in the most severe illnesses [27].

A recent study documented that leukaemia, thrombocytopenia, and anaemia could be used to predict malaria infections in regions where moderate parasitaemia predominates, particularly in low-resource settings [28]. Preventing malaria through vector control, insecticide-treated bed net deployment, early and precise illness diagnosis, and appropriate administration of potent antimalarial medications significantly lower the incidence of anaemia in tropical nations [15]. Severe thrombocytopenia raises the chance of dying from falciparum or vivax malaria, especially in people who also have profound anaemia [29]. The high rate of malaria morbidity and death is caused by the illness component of malaria, such as severe malarial anaemia, even though contemporary malaria management aims to eradicate the parasite. Malarial complications and unfavourable effects are prevented by early detection and timely treatment. Failure of antimalarial therapy might lead to higher transmission of malaria and accelerate drug resistance. Blood cell count parameters might be helpful during antimalarial treatment to track treatment progress and outcome [30]. Evaluating haematological parameters in malaria patients reduces hospital stays and adverse outcomes, especially in low-income countries, by facilitating early identification and improving treatment efficacy.

Limitation

The present study was a hospital-based study with limited cases. A further prospective study with a bigger sample size and a more extended follow-up period may help get a better outlook on the clinic-haematological spectrum of the disease in this zone of the country.

## Conclusions

Haematological abnormalities are frequently observed in acute falciparum malaria. The most common alterations were anaemia and thrombocytopenia. Most anaemia cases were normochromic and normocytic, a common morphologic presentation of RBCs in malarial anaemia. Less notable alterations were seen in the WBC. The coagulation parameters were elevated in severe malaria cases. The bone marrow has been connected to changes in cell maturation and cellularity. Inadequate erythropoietic response from the bone marrow to the associated anaemia was observed among malaria patients. A deeper understanding of the pathophysiology and host-parasite interactions is necessary to develop therapeutic options to treat severe malarial anaemia and gain more insight into the immunological mechanism involved in the malarial infection.

A severe degree of thrombocytopenia, anaemia, and multi-organ involvement among patients with hyperparasitaemia is associated with poor prognosis and mortality. Routine monitoring of patients suffering from malaria for haematological alterations and timely management of those conditions might help in reducing associated morbidity and mortality of the disease.
